# Knowledge and Awareness of Children’s Oral Health Among Parents in Taif, Saudi Arabia

**DOI:** 10.7759/cureus.109370

**Published:** 2026-05-21

**Authors:** Mohammed A Alzubaidi, Bandar S Shukr, Fahad S Algahtani, Amith HV, Muhannad Alamri, Rayan S Almalki, Mohanna M Alharthi, Talal Alotaibi, Abdullah Almalki, Mohammed Althagafi

**Affiliations:** 1 Department of Preventive Dentistry, Faculty of Dentistry, Taif University, Taif, SAU; 2 Department of Restorative Dental Science , Faculty of Dentistry, Taif University, Taif, SAU; 3 Public Health Dentistry, Triveni Institution of Dental Sciences, Hospital &amp; Research Center, Bilaspur, IND; 4 Faculty of Dentistry, Taif University, Taif, SAU

**Keywords:** dental caries, dental plaque, fluoride, oral hygiene practices, parental awareness, pediatric oral health, preventive dentistry, primary teeth, saudi arabia, taif

## Abstract

Objective: To assess parental knowledge and awareness of children’s oral health in Taif City, Saudi Arabia, and to identify demographic factors associated with preventive oral health practices.

Methods: A cross-sectional study was conducted using a self-administered Arabic questionnaire distributed electronically to parents of children aged 3-12 years in Taif City, Saudi Arabia. The survey assessed sociodemographic characteristics and parental knowledge and awareness of children’s oral health. Data were analyzed using descriptive and inferential statistics, including adjusted multiple linear regression analysis. Ethical approval was obtained from Taif University, and informed consent was obtained from all participants.

Results: A total of 352 parents participated (response rate: 100%). Most respondents had a college education or higher (61.1%). The overall mean oral health knowledge score was 5.38 ± 1.77 SD out of 10. Participants demonstrated the highest level of awareness regarding the relationship between oral and systemic health (94.9%), the definition of dental calculus (80.1%), the role of fluoride in caries prevention (70.7%), and the influence of primary teeth on permanent dentition (64.2%). In contrast, knowledge gaps were identified in the number of primary teeth (34.4%), the effect of plaque on gingival health (29.5%), toothbrush replacement frequency (22.4%), and the ideal brushing duration (51.4%). Significant associations with oral health awareness were found for monthly income (p = 0.023) and employment status (p = 0.004; 0.006), while education level and number of children did not significantly influence awareness.

Conclusion: Parents in Taif demonstrated moderate overall knowledge of pediatric oral health, with high awareness of oral-systemic health links and fluoride, but notable gaps in toothbrush replacement frequency, plaque understanding, and primary teeth awareness. Socioeconomic factors, particularly monthly income and employment status, significantly influenced parental oral health awareness. Targeted parental education programs addressing these specific knowledge gaps are needed to improve children's oral health outcomes.

## Introduction

Dental caries is one of the most prevalent chronic diseases affecting children worldwide and is closely associated with oral hygiene practices, dietary habits, fluoride exposure, and the timely adoption of preventive dental behaviors [1,2]. If left untreated, dental caries can lead to pain, infection, impaired mastication, and premature tooth loss, potentially affecting the development and eruption of permanent dentition and compromising children’s overall quality of life [3].

Parental knowledge and awareness play a crucial role in maintaining children’s oral health, as parents directly influence toothbrushing practices, dietary choices, fluoride use, and the utilization of preventive dental services [2,4]. Previous studies have demonstrated that children whose parents possess adequate oral health knowledge and positive attitudes toward preventive dentistry exhibit better oral hygiene behaviors and lower caries experience [3,5].

In Saudi Arabia, several studies have reported variability in parental awareness regarding optimal oral hygiene practices, the importance of primary teeth, and the appropriate timing of the first dental visit [4,6].Although preventive dental services are increasingly available, parental engagement with these services remains inconsistent. A study conducted in Riyadh demonstrated that, despite relatively high awareness of preventive dentistry concepts, the utilization of preventive procedures such as fluoride application and fissure sealants was limited and significantly associated with parental education and socioeconomic status [5,6].Similarly, research from Dammam revealed that a substantial proportion of parents sought dental care for their children only when pain was present, indicating poor adherence to preventive dental practices and limited awareness of fluoride-based preventive measures [1].

Socioeconomic status and parental education have been consistently identified as important determinants of children’s oral health. Studies conducted in Saudi Arabia and internationally have shown that higher parental education and income levels are associated with better oral health knowledge, improved preventive practices, and lower caries prevalence among children [2,5]. Additionally, misconceptions regarding the importance of primary teeth and concerns related to fluoride use have been reported among parents and may negatively influence preventive oral health behaviors [1,3].Despite the growing national emphasis on children’s oral health, data remain limited for mid-sized cities such as Taif, where socioeconomic and cultural characteristics may differ from those of larger metropolitan areas. This study aimed to assess parental knowledge and awareness regarding children's oral health within the Taif community, as well as to identify factors associated with preventive oral health practices, which is therefore essential to identifying local knowledge gaps and informing targeted preventive strategies aimed at improving early childhood oral health outcomes.

## Materials and methods

Study design and ethical considerations

A cross-sectional descriptive survey was conducted among parents of preschool- and school-aged children residing in Taif City, Saudi Arabia. Inclusion criteria were parents or legal guardians of children within the specified age range (3-12 years) who were able to read and understand Arabic, and who provided informed consent. Exclusion criteria included individuals who were not parents or legal guardians, parents of children outside the specified age range, and incomplete survey responses. Data collection was conducted after ethical approval, during November and December 2025. Ethical approval was obtained from the Scientific Research Ethics Committee, Taif University (Approval No. 47-070, dated November 2, 2025). Participation was voluntary, informed consent was obtained from all participants, and anonymity and confidentiality of data were ensured.

Study sample and data collection

A non-probability convenience sampling technique was used. According to the 2024 Ministry of Education report [7], the total population of preschool- and school-aged children was estimated to be 141,401 individuals. Using the Raoasft website, the sample size calculation was performed with a 95% confidence level and 5% margin of error. The estimated minimum sample size that should be included in this study was found to be 384 persons. In this study, a total of 400 individuals satisfied the inclusion criteria, agreed to participate, and completed the study questionnaire. However, 48 participants were excluded because of missing or incomplete survey responses; consequently, a total of 352 participants were included in the final study sample.

The study data were collected using an electronic survey link that was distributed via WhatsApp groups, social media platforms, and school communication channels to reach eligible participants. The structured, self-administered questionnaire was developed in Arabic and consisted of two sections (see Appendices). The first section collected demographic information, including educational level, household income, employment status, and number of children. The second section assessed parental knowledge and awareness of pediatric oral health and preventive practices, covering topics such as the number of primary teeth, recommended toothbrushing duration, toothbrush replacement frequency, timing of the first dental visit, understanding of dental plaque and calculus, the role of fluoride in caries prevention, and the relationship between oral and general health. The overall oral health knowledge score among the participating parents was calculated by summing the number of correct answers, with a minimum possible score of "zero" and a maximum score of "10".

Content validity of the questionnaire was established through expert review by specialists in dental public health, pediatric, and restorative dentistry. The questionnaire was pilot-tested on a subset of parents (n=20) to assess clarity and comprehension, and internal consistency reliability was evaluated using Cronbach’s alpha, with a value of 0.821 considered acceptable. The pilot-tested subjects were also excluded from the final study sample.

Statistical analysis

Survey responses were exported to IBM SPSS Statistics, Version 25.0 (IBM Inc., Armonk, NY, USA) for statistical analysis. Descriptive statistics, including frequencies, percentages, means, and standard deviations, were used to summarize the demographic characteristics and the questionnaire responses, as indicated. Moreover, inferential statistical analysis, specifically adjusted multiple linear regression, was conducted to examine the associations between different demographic variables and parental oral health awareness. Regression analysis findings were presented in 95% confidence intervals (CIs) and adjusted beta coefficients (Adj βs). Residual plots were created and evaluated to ensure that the variances’ homogeneity was satisfied. In addition, data normality was evaluated using histogram plots, as well as the Kolmogorov-Smirnov test. The variance inflation factor was utilized to identify any multicollinearity between the variables. Additionally, Cook's distance was used to detect notable outliers. The adjusted coefficient of determination (Adj. R²) was used to evaluate the model’s goodness of fit. All of these assumptions were satisfied in the model without any violations, with an acceptable model fitness (Adj. R² = 0.024). Furthermore, collapsing was done on groups with few individuals as needed. All statistical procedures were conducted using a two-tailed method, and statistical significance was considered at p≤0.05.

## Results

A total of 352 parents participated in the survey. Most were female (65.9%), aged 31-40 years (46.9%), held a bachelor’s degree or higher (61.1%), and had more than three children (42.9%). Regarding household income, 43.8% reported a monthly income of less than 4,000 Saudi Arabian Riyal (SAR), and 46.3% were employed in the governmental sector. Table 1 summarizes the demographic characteristics of the studied population.

**Table 1:** Demographic characteristics of the study participants (N=352) The data has been represented as frequency (n) and percentage (%).

Variable	Category	Frequency (n)	Percentage (%)
Sex	Male	120	34.1
	Female	232	65.9
Age	20 years or less	0	0.0
	21-30 years	134	37.2
	31-40 years	165	46.9
	41-50 years	43	12.2
	51 years or older	13	3.7
Education level	No education	8	2.3
	Elementary school	27	7.7
	Middle school	19	5.4
	High school	83	23.6
	College or higher	215	61.1
Monthly income (SAR)	<4,000	154	43.8
	4,000-10,000	73	10.7
	10,000-20,000	100	28.4
	>20,000	25	7.1
Occupation	Govermental sector	163	46.3
	Private sector	52	14.8
	Self-employed	9	2.6
	Unemployed	128	36.4
Number of children	One child	91	25.9
	Two children	60	17.0
	Three children	50	14.2
	More than three children	151	42.9

Overall, parental knowledge and awareness of pediatric oral health were moderate, with a mean correct response rate of 5.38 (±1.77 SD). Awareness was highest for the link between oral and general health (94.9%) and the distinction of dental calculus (80.1%). Knowledge of fluoride’s role in preventing caries, as well as the influence of primary teeth on permanent dentition, was moderate (70.7%, 64.2%, respectively). Almost half of the participating parents correctly identified the ideal duration for tooth brushing (51.4%), the ideal time for the first dental visit (46.0%), and characteristics of dental plaque (43.8%). Poor parental knowledge was observed among items related to the number of primary teeth (34.4%), the effect of plaque on gingival health (29.5%), and the frequency of toothbrush replacement (22.4%). Table 2 details participant responses to individual oral health knowledge questions.

**Table 2:** Summary of parental knowledge regarding pediatric oral health (N=352) The data has been represented as frequency (n) and percentage (%). Five out of 10 questions demonstrated high percentages of correct answers, indicating moderate parental awareness with notable gaps in specific domains.

Knowledge domain	Correct (n)	Correct (%)	Incorrect (n)	Incorrect (%)
Number of primary teeth	121	34.4	231	65.6
Ideal brushing duration	181	51.4	171	48.6
Toothbrush replacement frequency	79	22.4	273	77.6
Best age for first dental visit	162	46.0	190	54.0
Definition of dental plaque	154	43.8	198	56.2
Definition of dental calculus	282	80.1	70	19.9
Importance of fluoride	249	70.7	103	29.3
Effect of plaque on gingival health	104	29.5	248	70.5
Primary teeth influence on permanent	226	64.2	126	35.8
Relationship between oral and general	334	94.9	18	5.1

The associations between demographic variables and parental overall oral health knowledge are presented in Table 3. The findings indicated a significant association with sex (p-value = 0.031), monthly income (p-value = 0.023), as well as employment status (p-value = 0.004; p-value = 0.006). Female parents had an average oral health knowledge that was 0.444 points lower than that of male parents. Similarly, lower levels of knowledge were observed among those who were employed, specifically in governmental (1.038 points) and private (0.964 points) sectors. On the other hand, parents who received a monthly income of 4,000 SAR or more showed higher average knowledge by 0.768 points compared to those who received less. No significant effects were observed for the remaining demographic variables (i.e., age, education level, and number of children) on the parents’ average overall oral health knowledge.

**Table 3:** Association between demographic factors and oral health awareness (N=352) The data have been analyzed using an adjusted multiple linear regression model (model Adj. R² = 0.024), and presented as 95% confidence intervals (CIs) and adjusted beta coefficients (Adj β). * Statistical significance set at (p<0.05), indicating that sex, monthly income, and employment status were significantly associated with parental oral health awareness. SAR: Saudi Arabian Riyal

Demographic variable	Categories	Adj β	95% CI	P-value
Sex	Male	Reference	Reference	Reference
	Female	-0.444	-0.848, -0.040	0.031*
Age	30 years or less	Reference	Reference	Reference
	31 - 40 years	-0.277	-0.709, 0.156	0.209
	41 years or older	-0.052	-0.612, 0.509	0.857
Education level	High school or less	Reference	Reference	Reference
	College or higher	-0.052	-0.447, 0.343	0.796
Monthly income (SAR)	<4,000	Reference	Reference	Reference
	≥4,000	0.768	0.108, 1.428	0.023*
Occupation	Self-employed / Unemployed	Reference	Reference	Reference
	Governmental sector	-1.038	-1.739, -0.336	0.004*
	Private sector	-0.964	-1.647, -0.282	0.006*
Number of children	Three children or less	Reference	Reference	Reference
	More than three children	0.146	-0.239, 0.531	0.456

## Discussion

This study assessed parental knowledge and awareness regarding children's oral health and factors affecting preventive oral health practices in Taif, Saudi Arabia. The overall mean knowledge score of 5.38 ± 1.77 SD out of 10 indicates a moderate level of parental oral health awareness, with notable variability across knowledge domains. High awareness of the oral-systemic health relationship (94.9%) is consistent with findings from Riyadh and Dammam, where similar recognition was reported [6,8]. Despite this, substantial gaps persist in foundational concepts. Only 34.4% of parents correctly identified the number of primary teeth, and 22.4% knew the recommended toothbrush replacement frequency. Comparable deficiencies in basic oral health knowledge have been reported in Riyadh and Abha, suggesting a nationwide trend [1,3].Furthermore, only 46.0% recognized the ideal age for the first dental visit, consistent with findings from Dammam indicating delayed awareness of early dental attendance [9].

Parents demonstrated stronger knowledge regarding fluoride (70.7%) and calculus (80.1%), echoing patterns observed in Riyadh [6].However, awareness of plaque-related gingival disease was low (29.5%), similar to previous Saudi studies reporting limited parental understanding of microbial dental concepts, which are less visible and often overlooked [1,3]. Socioeconomic factors significantly influenced parental oral health knowledge. Parents earning ≥4,000 SAR monthly demonstrated significantly higher awareness (Adj β = 0.768; p = 0.023) compared to those earning less, while those employed in governmental and private sectors showed lower awareness (Adj β = −1.038 and −0.964, respectively; p = 0.004 and 0.006). Education level and number of children did not significantly influence oral health awareness (p = 0.796 and 0.456). These findings align with multiple Saudi studies identifying parental socioeconomic status as a key determinant of oral health knowledge and child oral health outcomes, underscoring the need for targeted preventive programs for lower-income and formally employed populations [4,5].

In the present study, parents in Taif showed moderate overall oral health knowledge, with good awareness of oral-systemic links and fluoride, but clear gaps in toothbrush replacement frequency, plaque recognition, and awareness of primary teeth. Similar findings have been reported in Riyadh and the western region, where parental knowledge was described as acceptable overall, but important gaps persisted in early recognition of caries, understanding of bacterial transmission, and awareness of professionally applied preventive measures, and many parents sought dental care only in response to pain or trauma [4,8,10].In Jeddah, less than one‑third of parents correctly identified the recommended age for the first dental visit, and routine check‑ups were uncommon, mirroring the knowledge-practice gap observed in Taif [9]. Together, these studies suggest that many Saudi parents recognize the importance of oral health but do not consistently translate this awareness into optimal preventive behaviors, particularly in relation to early dental visitation and effective toothbrushing practices [9,10].

International evidence supports this pattern. Parents in India and Brazil have demonstrated reasonable awareness of caries and the importance of primary teeth, yet showed limited understanding of the ideal age for the first dental visit and did not always implement recommended preventive routines [11,12]. In Brazil, higher parental oral health awareness was associated with previous dental visits among preschool children, underscoring the combined influence of awareness and socioeconomic factors on service utilization [12]. These findings are consistent with the present study, where higher income was associated with better knowledge scores, indicating that parents with lower income and those in formal employment should be prioritized in oral health promotion efforts in Taif. From a public health perspective, these results suggest that generic awareness campaigns may be insufficient and should be complemented by targeted interventions focusing on toothbrush replacement frequency, plaque control, feeding practices, and adherence to the recommendation for the first dental visit [4,9].School‑based activities, oral‑health counseling integrated into pediatric and primary‑care visits, and simple, culturally appropriate educational materials may help narrow the knowledge-practice gap in this population [4,9,11].In clinical practice, dentists and hygienists in Taif can play a central role by offering structured parental counseling on early dental attendance, supervised twice‑daily brushing with fluoridated toothpaste, and limiting sugary snacks, with particular attention to families of lower socioeconomic status and those in formal employment [4,9,10,12].

The current study has some limitations. First, because the study is cross-sectional in nature, it is difficult to establish causal associations between the various demographic parameters and parental knowledge. Second, the non-randomized convenience sampling technique used in this study may have impacted the generalizability of the study's findings because it is not an accurate representation of the target population. Third, recall bias is expected to affect the study findings due to the use of self-reported survey questions for collecting the research data. Fourth, selection bias is expected to affect the study findings due to the use of online channels to distribute the study survey, which, in most cases, did not reach all the targeted parents in the population. Finally, although some demographic associations reached statistical significance (i.e., sex, monthly income, occupation), the practical predictive value of the model (Adj. R²=0.024) remains limited, and there may be additional unmeasured factors that could contribute to parental oral health knowledge in the study population. These findings highlight the need for tailored oral health education programs in Taif, focusing on toothbrush replacement, plaque control, and early dental visits. School-based initiatives, pediatric dental clinics, and primary healthcare centers may serve as effective platforms for dissemination. Future research should examine whether enhanced parental knowledge improves clinical outcomes in children, and evaluate the impact of community-based interventions in mid-sized Saudi cities such as Taif.

## Conclusions

Parents in Taif exhibit moderate knowledge and awareness of children's oral health, with gaps in toothbrush replacement frequency, plaque recognition, and primary teeth awareness. Socioeconomic factors, specifically monthly income and employment status, significantly influence parental oral health awareness, while education level and number of children did not demonstrate significant associations. Targeted education programs through schools, clinics, and community health initiatives are needed to improve parental knowledge and support better oral health outcomes in children, with particular emphasis on lower-income families and those in formal employment sectors.

## Appendices

**Table 4:** The study questionnaire

Section	Sentence in the questionnaire	Answer options
Demographics	What is your sex?	Male
		Female
	What is your age?	20 years or less
		21 - 30 years
		31 - 40 years
		41 - 50 years
		51 years or older
	What is your education level?	No education
		Elementary school
		Middle school
		High school
		College or higher
	What is your monthly income (SAR)?	<4,000
		4,000–10,000
		10,000–20,000
		>20,000
	What is your occupation?	Governmental sector
		Private sector
		Self-employed
		Unemployed
	How many children do you have?	One child
		Two children
		Three children
		More than three children
Parental knowledge regarding pediatric oral health	How many baby teeth are there in children?	12 teeth
		16 teeth
		20 teeth
	How long is it appropriate to brush the teeth?	Few seconds
		1 minute
		2 minutes
		3 minutes
	When should you replace your toothbrush?	Every month
		Every 3 months
		Every year
		Replacement is not needed
	What is the best age for a child to make their first visit to the dentist?	At birth
		6 months - 1 year
		After 6 years
		When feeling pain
	What does dental plaque mean?	A soft layer that forms on the teeth
		A hard layer that forms on the teeth
		I don’t know
	What does dental calculus mean?	A soft layer that forms on the teeth
		A hard layer that forms on the teeth
		I don’t know
	How does dental plaque affect the tooth?	It causes gum disease
		It causes bad breath
		It causes tooth discoloration
		I don’t know
	Do baby tooth problems affect permanent teeth?	Yes
		No
		I don’t know
	Do you think a child's oral health affects his or her overall health?	Yes
		No
		I don’t know
